# PB.36. Imaging in gynaecomastia: audit of referral and imaging practise to establish referral guidelines

**DOI:** 10.1186/bcr3718

**Published:** 2014-11-03

**Authors:** K Bonam, A Leaver, S Sharma

**Affiliations:** 1 Gateshead Hospitals NHS Trust, Gateshead, UK

## Introduction

There is variation in the literature and between breast units in use of imaging in clinically suspected gynaecomastia. Our study aim was to audit referral and imaging in our breast unit, formulating guidelines and standardising practise.

## Methods

The authors performed retrospective data collection of imaging and pathology for clinically suspected gynaecomastia in our breast unit between January 2012 and May 2014, using hospital radiology and pathology computer information systems. Data included clinical history, clinical score (P1 to P5), ultrasound (U1 to U5) and mammographic scores (M1 to M5). Patients with clinical suspicion of malignancy (written on imaging request or P4/P5) were excluded. Descriptive statistics were performed.

## Results

A total of 177 male patients had been referred for imaging with P1 (19), P2 (113), P3 (11) or no P value (34), clinical history stating or querying gynaecomastia. Ultrasound was performed on all patients. All P1 and P2 patients were U1 or U2, and M1 or M2 if mammogram was performed (0/19 P1 patients, 20/113 P2 patients, 4/11 P3 patients). In P1, P2 and no P value patients, 6/166 patients underwent ultrasound-guided biopsy (5/6 B2 gynaecomastia, 1/6 B1 benign angiolipoma). In P3 patients, two biopsies were performed: B2 gynaecomastia in one patient, but one biopsy (M4U4) revealed B5b invasive carcinoma.

## Conclusion

Imaging confirmed the clinical findings in all P1 and P2 patients, but a clinically unsuspected malignancy was diagnosed through imaging of a P3 patient. The findings support guideline formulation where imaging is not indicated for typical (P1, P2) gynaecomastia. Imaging should, however, be performed where there is any doubt at all about the clinical diagnosis.

